# Mo_*x*_C Heterostructures as Efficient Cocatalysts in Robust Mo_*x*_C/g-C_3_N_4_ Nanocomposites for Photocatalytic H_2_ Production from Ethanol

**DOI:** 10.1021/acssuschemeng.3c06261

**Published:** 2024-03-07

**Authors:** Yan Wang, Arturo Pajares, Jarosław Serafin, Xavier Alcobé, Frank Güell, Narcís Homs, Pilar Ramírez de la Piscina

**Affiliations:** † Departament de Química Inorgànica i Orgànica, secció de Química Inorgànica & Institut de Nanociència i Nanotecnologia (IN2UB), Universitat de Barcelona, Martí i Franquès 1, 08028 Barcelona, Spain; ‡ Catalonia Institute for Energy Research (IREC), Jardins de les Dones de Negre 1, 08930 Barcelona, Spain; § Unitat de Difracció de Raigs X, Centres Científics i Tecnològics (CCiTUB), Universitat de Barcelona, Lluís Solé i Sabaris 1-3, 08028 Barcelona, Spain; ∥ ENPHOCAMAT Group, Institut de Nanociència i Nanotecnologia (IN2UB), Universitat de Barcelona, Martí i Franquès 1, 08028 Barcelona, Spain

**Keywords:** molybdenum carbide, H_2_ photoproduction, visible photocatalysis, Mo_*x*_C cocatalyst, g-C_3_N_4_, bioethanol

## Abstract

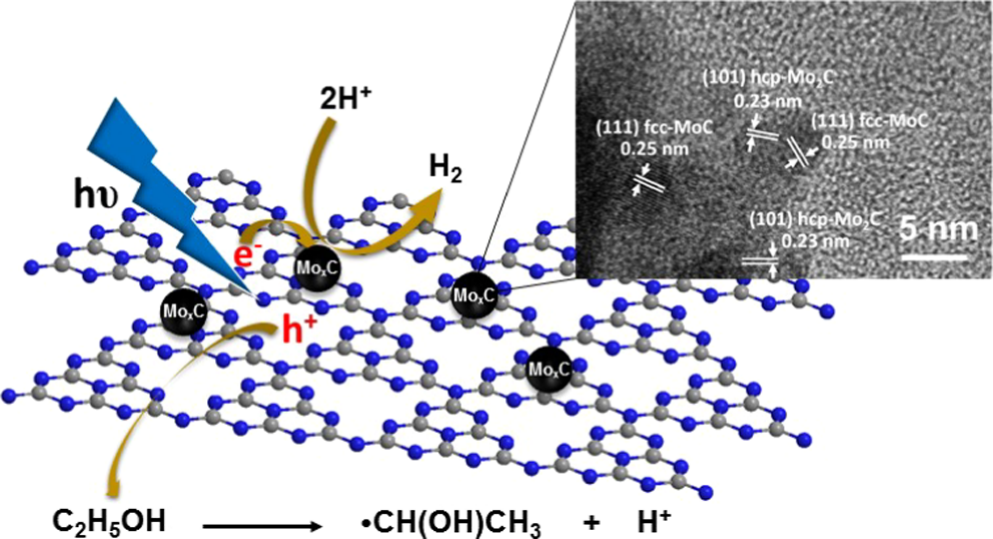

In this work, we studied new materials free of noble metals that are active in photocatalytic H_2_ generation from ethanol aqueous solutions (EtOH_aq_), which can be obtained from biomass. Mo_*x*_C/g-C_3_N_4_ photocatalysts containing hexagonal (hcp) Mo_2_C and/or cubic (fcc) MoC nanoparticles on g-C_3_N_4_ nanosheets were prepared, characterized, and evaluated for photocatalytic hydrogen production from EtOH_aq_ (25% v/v). Tailored Mo_*x*_C/g-C_3_N_4_ nanocomposites with Mo_*x*_C crystallite sizes in the 4–37 nm range were prepared by treatment with ultrasound of dispersions containing Mo_*x*_C and g-C_3_N_4_ nanosheets, formerly synthesized. The characterization of the resulting nanocomposites, Mo_*x*_C/g-C_3_N_4_, by different techniques, including photoelectrochemical measurements, allowed us to relate the photocatalytic performance of materials with the characteristics of the Mo_*x*_C phase integrated onto g-C_3_N_4_. The samples containing smaller hcp Mo_2_C crystallites showed better photocatalytic performance. The most performant nanocomposite contained nanoparticles of both hcp Mo_2_C and fcc MoC and produced 27.9 mmol H_2_ g^–1^ Mo; this sample showed the lowest recombination of photogenerated charges, the highest photocurrent response, and the lowest electron transfer resistance, which can be related to the presence of MoC-Mo_2_C heterojunctions. Moreover, this material allows for easy reusability. This work provides new insights for future research on noble-metal-free g-C_3_N_4_-based photocatalysts.

## Abstract 2

The Mo_*x*_C characteristics drive photocatalytic H_2_ production from ethanol under visible light irradiation using noble-metal-free Mo_*x*_C/g-C_3_N_4_ nanocomposites.

## 1 Introduction

Nowadays, the large-scale industrial production of H_2_ is captive to fossil fuels. Thus, new ways to produce H_2_ are of current interest in both the energy and environmental contexts. From this perspective, the development of photocatalytic systems that produce H_2_ using renewable substrates, such as bioalcohols, is of primary importance.^1−4^ Photocatalysts are usually based on inorganic semiconductor materials, and the direct use of solar radiation is highly attractive when the photocatalyst is able to absorb visible light. In this context, one of the most encouraging materials is graphitic carbon nitride (g-C_3_N_4_).^5−11^ On the other hand, the main concern related to the efficiency of photocatalysts is the usually fast rate of recombination of the photogenerated charges, electrons (e^–^), and holes (h^+^).^12^ Although the most common strategy that is used for improving h^+^/e^–^ charge separation is the addition of noble metals to the surface of the semiconductor, other compounds such as transition metal oxides, sulfides, or carbides, have been also proposed as efficient cocatalysts.^13,14^ Specifically, the catalytic properties of molybdenum and tungsten carbides are oftentimes described as Pt-like and they have been used as catalysts in different electrochemical and chemical processes and as cocatalysts in photochemical processes.^15−26^ The use of molybdenum carbides in alternative hydrogen production, has been mainly focused on their use as electrocatalysts.^14^ However, an increasing interest exists in the use of molybdenum carbide-based systems as cocatalysts in the photoproduction of H_2_.^14^ Different photocatalysts for H_2_ evolution containing molybdenum carbides and semiconductors, such as TiO_2_, CdS, and SrTiO_3_, have been reported.^14,27−30^ However, a limited number of papers dealing with photocatalysts based on Mo_2_C and g-C_3_N_4_ have been published.^31−35^ Mo–Mo_2_C/g-C_3_N_4_ and Co-doped Mo–Mo_2_C/g-C_3_N_4_,^31,35^ Mo_2_C@C/g-C_3_N_4_ heterostructures,^32,33^ and rod-like g-C_3_N_4_ decorated with Mo_2_C,^34^ have been studied as photocatalysts in H_2_ generation; in every case, triethanolamine (TEOA) solution was employed as sacrificial electron donor in the photocatalytic test. The presence of Mo, Co and Mo or C enhances the cocatalyst effect of Mo_2_C onto g-C_3_N_4_.^31−35^ On the other hand, it has been reported that the presence of MoC/Mo_2_C heterostructures has a positive effect in the electrocatalytic behavior of molybdenum carbide-based catalysts for the H_2_ evolution reaction.^36^ Moreover, it has been proposed that, for a given material, there is a relationship between its performance in the electrocatalytic H_2_ evolution and its efficiency as a cocatalyst in the photocatalytic H_2_ production.^35^

This background and the advantage of using ethanol, which is currently produced and used as a biofuel around the world, led us to an in-depth study of photocatalysts based on Mo_*x*_C/g-C_3_N_4_ (Mo_*x*_C = Mo_2_C and/or MoC) for H_2_ production using EtOH_aq_ and visible light. We have recently reported the preparation of molybdenum carbide phases via sol–gel methods using different molybdenum precursors and carbon sources; Mo_2_C and/or MoC cubic and/or hexagonal were obtained depending on the preparation method used.^24,37−39^ In this work, we report the preparation and characterization of new tailored Mo_*x*_C/g-C_3_N_4_ photocatalysts, containing hcp Mo_2_C and/or fcc MoC as cocatalysts with different crystallite sizes. Hydrogen production is related to the photoelectrochemical properties of the nanocomposites, which in turn depend on the crystalline phases of molybdenum carbide, including charge recombination, electron transfer resistance, and the photocurrent response of different photocatalysts.

## 2 Experimental Section

### 2.1 Synthesis of Mo_*x*_C

Different Mo_*x*_C phases were synthesized based on a recently described sol–gel method using MoCl_5_ (reagent grade, 95%) and 4,5-dicyanoimidazole (DI) (reagent grade, 99%) from Sigma-Aldrich, as Mo and C sources, respectively.^37,38^

Specifically, the gel formed by the addition of MoCl_5_ (5.6 mmol) and DI (2.8 mmol) to 15 mL of ethanol (>99.999% HPLC, Sigma-Aldrich), was treated at different temperatures under an Ar flow (99.999%, Linde) (*T* = 700, 800, or 900 °C) in a tubular furnace. The materials were labeled Mo_*x*_CT, where *T* is the temperature used in the treatment. The preparation of Mo_*x*_C1100 was accomplished using a similar method, with MoCl_5_ (5.6 mmol), DI (5.6 mmol), and a thermal treatment of 1100 °C.

### 2.2 Preparation of g-C_3_N_4_ Nanosheets

For the preparation of g-C_3_N_4_ nanosheets, the thermal polymerization of melamine was accomplished as follows:^40^ melamine (>99%, Alfa Aesar) was calcined at 5 °C/min up to 520 °C (4 h); the yellowish material was ground to powder, and the calcination process was repeated.

### 2.3 Integration of Mo_*x*_CT onto g-C_3_N_4_

For the preparation of Mo_*x*_CT/g-C_3_N_4_, dispersions in ethanol with the appropriate amounts of Mo_*x*_CT and g-C_3_N_4_, to obtain about 3 wt% Mo, were treated under ultrasound (SONICS VCX 500) at 20 °C and 250 W for 1 h; then, ethanol was eliminated by careful evaporation with stirring at 50 °C. Moreover, using a similar procedure, commercial Alfa-Aesar Mo_2_C (99.5% metal basis, hcp Mo_2_C, average crystallite size *d̅* = 37 nm), was used for the preparation of the Mo_2_C-comm/g-C_3_N_4_ photocatalyst. Table 1 lists all of the photocatalysts prepared and studied in this work.

**Table 1:** Several Characteristics of Photocatalysts^a^

			002		
sample	Mo content (wt %)	BET (m^2^/g)	g-C_3_N_4_ (2θ deg)	crystallite size (nm)	band gap (eV)
Mo_*x*_C700/g-C_3_N_4_	3.65	39	27.7	11 (Mo_2_C)^b^	2.79
				4 (MoC)^b^	
Mo_2_C800/g-C_3_N_4_	3.43	34	27.7	23 (Mo_2_C)	2.79
Mo_2_C900/g-C_3_N_4_	3.35	39	27.6	31 (Mo_2_C)	2.78
MoC1100/g-C_3_N_4_	2.62	22	27.5	4 (MoC)	2.77
Mo_2_C-comm/g-C_3_N_4_	3.33	22	27.5	37 (Mo_2_C)	2.77
g-C_3_N_4_		31	27.7		2.75

a Mo content from chemical analysis (ICP-AES), BET surface area, position of the 002 XRD peak of g-C_3_N_4_, Mo_*x*_C phase, crystallite size, and band gap values.

b Calculated from the full profile analysis of the XRD pattern (Figure S6 ^43^).

### 2.4 Characterization of Photocatalysts

Inductively coupled plasma atomic emission spectrometry (ICP-AES) was used to determine the Mo content using a PerkinElmer Optima 3200RL instrument.

A Micromeritics Tristar II 3020 instrument was used to record the N_2_ adsorption–desorption isotherms, which were determined at −196 °C after degasification at 250 °C under an Ar flow. Multipoint Brunauer–Emmett–Teller (BET) analysis of the isotherms was used to calculate the specific surface area (*S*_BET_). The desorption isotherms were used for the determination of the pore size distribution by the method of Barrett–Joyner–Halenda (BJH).

Powder X-ray diffraction (XRD) patterns were recorded using a Bragg–Brentano powder diffractometer (PANalytical X’Pert PRO MPD), with Cu Kα radiation (λ = 1.5418 Å) from 2θ = 4 to 100°. Peak indexation and phase identification were performed with the aid of the ICDD Powder Diffraction File (PDF).^41^ Accurate peak positions, area intensities, and full width at half-maximum (fwhm) were obtained after full profile analysis. The crystallite sizes were calculated from the fwhm using the Scherrer equation.^42^ Semiquantitative phase analysis, in some binary nanocrystalline samples, was performed from the accurate area intensities and using the reference intensity ratios in the corresponding PDF file.^43^

Fourier-transform infrared spectroscopy (FTIR) analysis was conducted by using pellets of the KBr-diluted samples on a Thermo Nicolet 5700 FTIR apparatus.

Transmission and high-resolution electron microscopy (TEM-HRTEM) and energy-dispersive X-ray (EDX) spectroscopy with elemental mapping analysis were performed using a JEOL JEM-2100 instrument operating at 200 kV.

X-ray photoelectron spectroscopy (XPS) was conducted using a PerkinElmer PHI-5500 Multitechnique System with Al Kα radiation (1486.6 eV). The binding energy (BE) values were determined using the C 1s peak at 284.8 eV, which was previously ascertained using Au as a reference. MultiPak XPS software was used to deconvolute the XPS signals.

UV–vis diffuse reflectance spectra (UV–vis DRS) were acquired using a PerkinElmer Lambda 950 UV/vis Spectrometer; BaSO_4_ was used as a reference. The Kubelka–Munk model was used to determine the band gap values.

Photoluminescence (PL) spectroscopy measurements were performed at room temperature by using a Kimmon IK Series He–Cd CW laser (325 nm, 40 mW). Fluorescence was dispersed through a SpectraPro 2750 (focal length of 750 mm) f/9.8 monochromator, detected with a Hamamatsu H8259-02 photomultiplier, and amplified using a Stanford Research System SR830 DSP Lock-in amplifier. A 360 nm filter was used for the stray light, and the emission spectra were corrected using the optical transfer function of the PL setup.

For photoelectrochemical characterization of samples, electrochemical impedance spectroscopy (EIS) and transient photocurrent determination were performed. The system consisted of a computer-controlled potentiostat (VMP3, BioLogic Science Instruments) with an undivided three-electrode cell; a Pt wire was used as the counter electrode, Ag/AgCl (3 M KCl) as the reference, the photocatalyst (1 cm^2^ geometric area) as the working electrode; and an aqueous solution of Na_2_SO_4_ (0.5 M) as the electrolyte. A150 W AM 1.5G solar simulator (Solar Light Co., 16S-300-002 v 4.0) with an incident light intensity of 1 sun (100 mW cm^–2^) was used to perform the measurements under illumination.

### 2.5 Photocatalytic Experiments

Figure S1 shows a schematic diagram of the system used for the photocatalytic tests. It contained a jacketed reactor of 300 mL, operated under continuous gas flow, and was equipped with a condenser, kept at −15 °C at the outlet.^44^ A broad-spectrum commercial (ACE-Hanovia) Hg lamp was used (Figure S1), which was immersed in the solution inside a water-cooled jacket that served as a UV cut-off filter (λ > 385 nm). The experiments were performed at atmospheric pressure and 20 °C. Before the photocatalytic test, the photocatalyst (300 mg) was dried at 100 °C, and EtOH_aq_ (250 mL, 25% v/v) was purged with Ar; and the corresponding suspension was stirred in the reactor for 30 min under dark and N_2_/Ar flow (99.999% N_2_ was used as the internal standard) and then irradiated. No products were detected in the dark. After 10 min of light on, the evolved gaseous products were periodically analyzed online using a gas microchromatograph Varian CP-4900, equipped with micro-TCD detectors (detection limit for H_2_, 50 ppm) and two columns (10 m PPQ, He carrier; 10 m molecular sieve (5 Å), Ar carrier).

After the test (4 h), the mixture was filtered, and the solution was analyzed by gas chromatography using Bruker 450 GC equipment with an FID detector and a CP-Sil 8 CB capillary column (30 m × 0.25 mm).

A reusability test was carried out with the most active photocatalyst; the used sample was removed from the liquid reaction mixture by filtering, washed with ethanol, and tested again. Moreover, in a separate cyclic experiment, the light was switched off after 1 h, and then after 0.5 h in dark conditions, the light was switched on for 1 h.

## 3 Results and Discussion

### 3.1 Structural and Chemical Characterization

As stated in the Experimental Section, the Mo_*x*_CT/g-C_3_N_4_ catalysts studied in this work were prepared by ultrasonic treatment of a suspension containing previously synthesized molybdenum carbide nanoparticles and g-C_3_N_4_ nanosheets. XRD patterns of the initial Mo_*x*_C800 and Mo_*x*_C900 (Figure S2) show the main presence of relatively narrow peaks that could be perfectly indexed with the hexagonal close-packed, hcp, *P*6_3_/*mmc*, structure of Mo_2_C (PDF 04-014-1517). The average crystallite size of Mo_2_C was *d̅* = 22 and 30 nm in Mo_*x*_C800 (from now Mo_2_C800) and Mo_*x*_C900 (from now Mo_2_C900), respectively. On the other hand, in the XRD pattern of Mo_*x*_C1100 (Figure S2), mainly 6 very wide peaks are observed that could be in principle be well indexed as the 111, 200, 220, 311, 222, and 400 reflections of the cubic close-packed, fcc, *Fm*3̅*m*, the structure of MoC (PDF 04-003-1480) with *d̅* = 4 nm; for easy identification of this sample, from this point on it will be labeled MoC1100. Meanwhile, the XRD analysis of Mo_*x*_C700 (Figure S2) shows both the presence of very wide peaks of the fcc structure of MoC and less wide peaks of the hcp structure of Mo_2_C.

**Figure 1:**
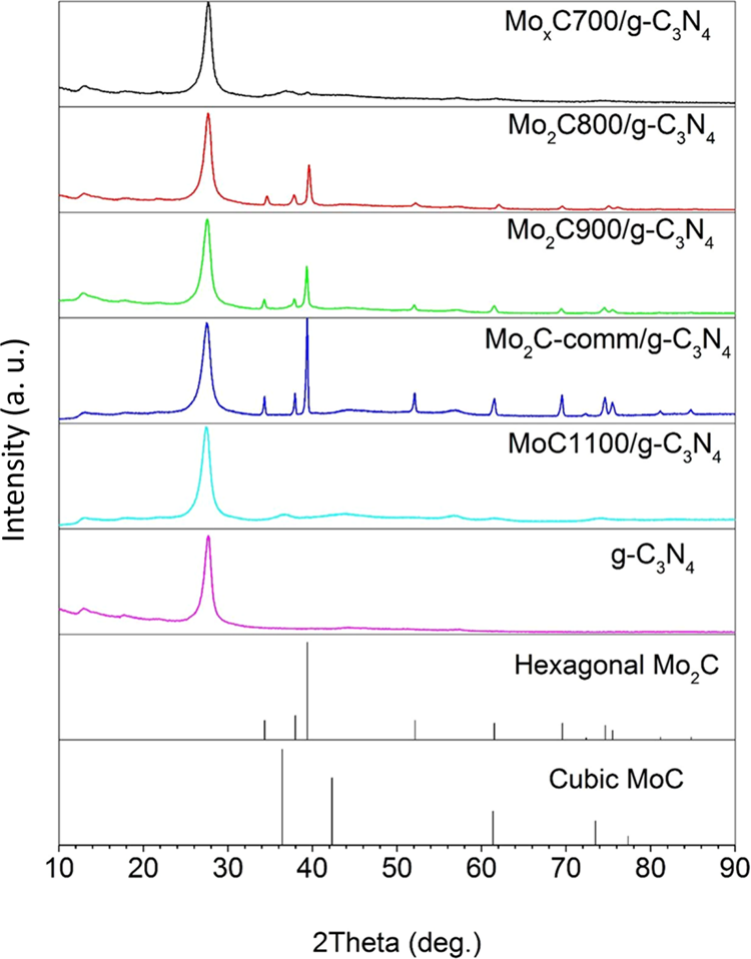
XRD patterns of nanocomposites and g-C_3_N_4_.

The XRD pattern of g-C_3_N_4_ prepared in this work shows a main peak at 2θ = 27.7° and a peak at 2θ = 13.0° with a much lower intensity (Figure 1). The peak at 2θ = 27.7° is due to 002 reflection of the (001) interlayer stacking; the position of this peak indicates a slightly smaller interlayer distance with respect to bulk g-C_3_N_4_, which shows the 002 XRD peak at 27.34°.^40,45^ The low intensity peak at 2θ = 13.0° is attributed to the in-plane structural packing motifs.^46^

The FTIR spectrum of g-C_3_N_4_ (Figure S3) agrees with that expected for this material.^40,45^ The characteristic band of the breathing mode of s-triazine rings can be seen at 807 cm^–1^; bands related to C=N and C–N stretchings in the heterocycles are visible in the 1800–900 cm^–1^ zone.^40,45^ All of these FTIR features can also be seen in the spectra of all nanocomposites prepared (Figure S3).

All photocatalysts showed type IV N_2_ adsorption–desorption isotherms with H3 hysteresis loops (Figure S4).^47^ The *S*_BET_ values of 29–39 m^2^ g^–1^ (Table 1), and wide pore size distributions in the range of meso- and macropores were found (Figure S5).

Figure 1 shows the XRD patterns of Mo_*x*_CT/g-C_3_N_4_ nanocomposites. After the integration of Mo_*x*_CT onto g-C_3_N_4_, the photocatalysts always retained the initial Mo_*x*_C crystalline phases and g-C_3_N_4_ nanosheets used in their preparation (Figure 1).

For Mo_2_C900/g-C_3_N_4_, MoC1100/g-C_3_N_4_, and Mo_2_Ccomm/g-C_3_N_4_, the 002 interlayer-stacking peaks appeared at slightly a lower angle than that of the prepared g-C_3_N_4_ (2θ = 27.7°) (Table 1).

Table 1 shows the obtained crystallite sizes of the Mo_*x*_C phases in the photocatalysts. The semiquantitative Mo_*x*_C phase analysis of the Mo_*x*_C700/g-C_3_N_4_ sample results in hcp Mo_2_C (14%) and fcc MoC (86%).^43^

Hexagonal Mo_2_C (11–31 nm crystallite size) was identified in the Mo_*x*_CT/g-C_3_N_4_ (*T* = 700–900 °C) nanocomposites; the higher the temperature used in the preparation of Mo_*x*_CT, the larger the Mo_2_C crystallite size. Mo_2_C-comm/g-C_3_N_4_ showed the largest hcp Mo_2_C crystallite size (37 nm). On the other hand, the fcc MoC phase, which was determined in Mo_*x*_C700/g-C_3_N_4_ and MoC1100/g-C_3_N_4_, showed a similar crystallite size (4 nm) in both nanocomposites.

**Figure 2:**
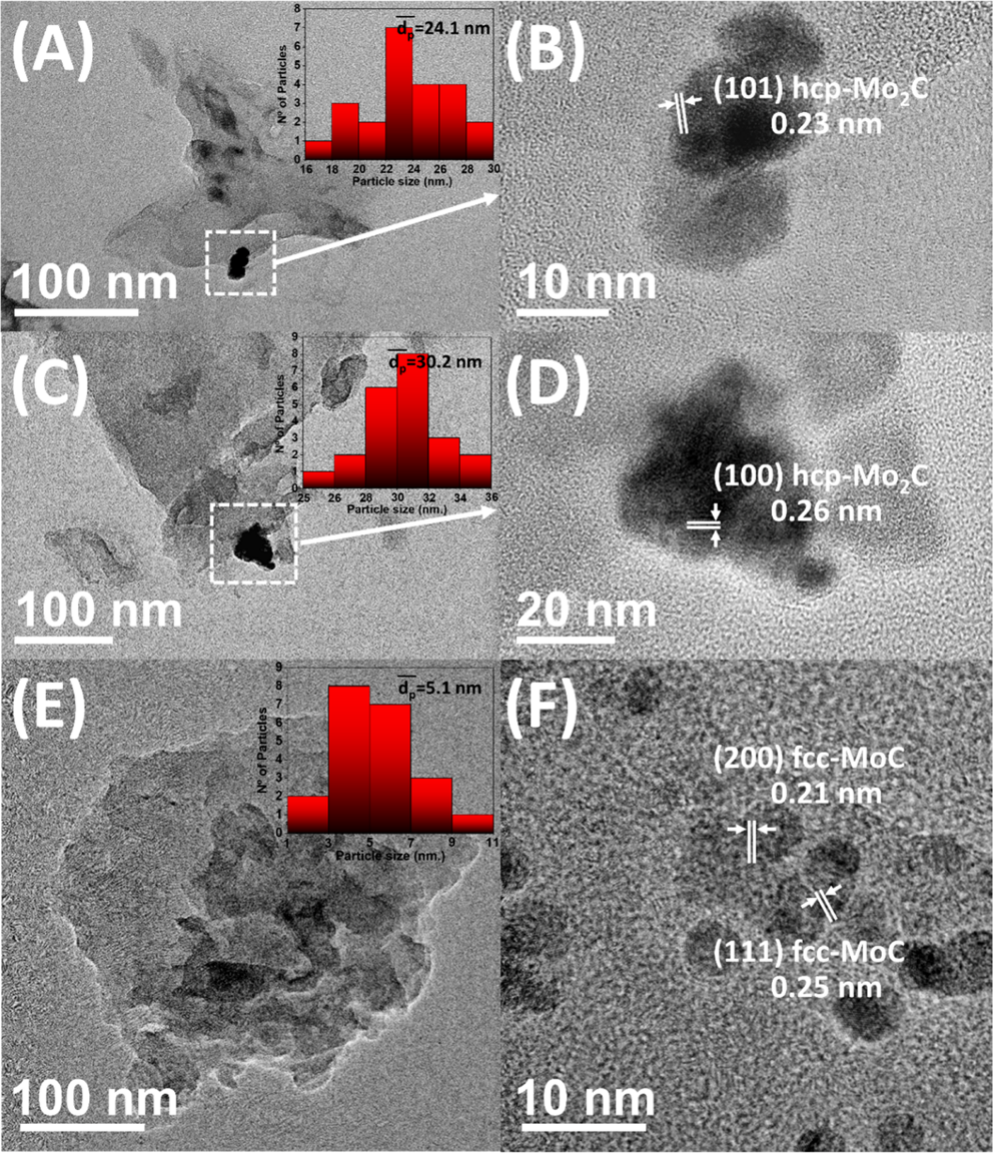
TEM-HRTEM images and particle distribution (insets) of (A) and (B) Mo_2_C800/g-C_3_N_4_; (C) and (D) Mo_2_C900/g-C_3_N_4_; and (E) and (F) MoC1100/g-C_3_N_4_ nanocomposites.

Figure 2 displays TEM-HRTEM images of the Mo_2_C800/g-C_3_N_4_, Mo_2_C900/g-C_3_N_4_, and MoC1100/g-C_3_N_4_ photocatalysts; hcp Mo_2_C nanoparticles were identified in Mo_2_C800/g-C_3_N_4_ (mean size: 24.1 nm) and Mo_2_C900/g-C_3_N_4_ (mean size: 30.2 nm), whereas cubic MoC nanoparticles were found in MoC1100/g-C_3_N_4_ (mean particle size: 5.1 nm). These findings agree with those determined by XRD analysis (Table 1).

**Figure 3:**
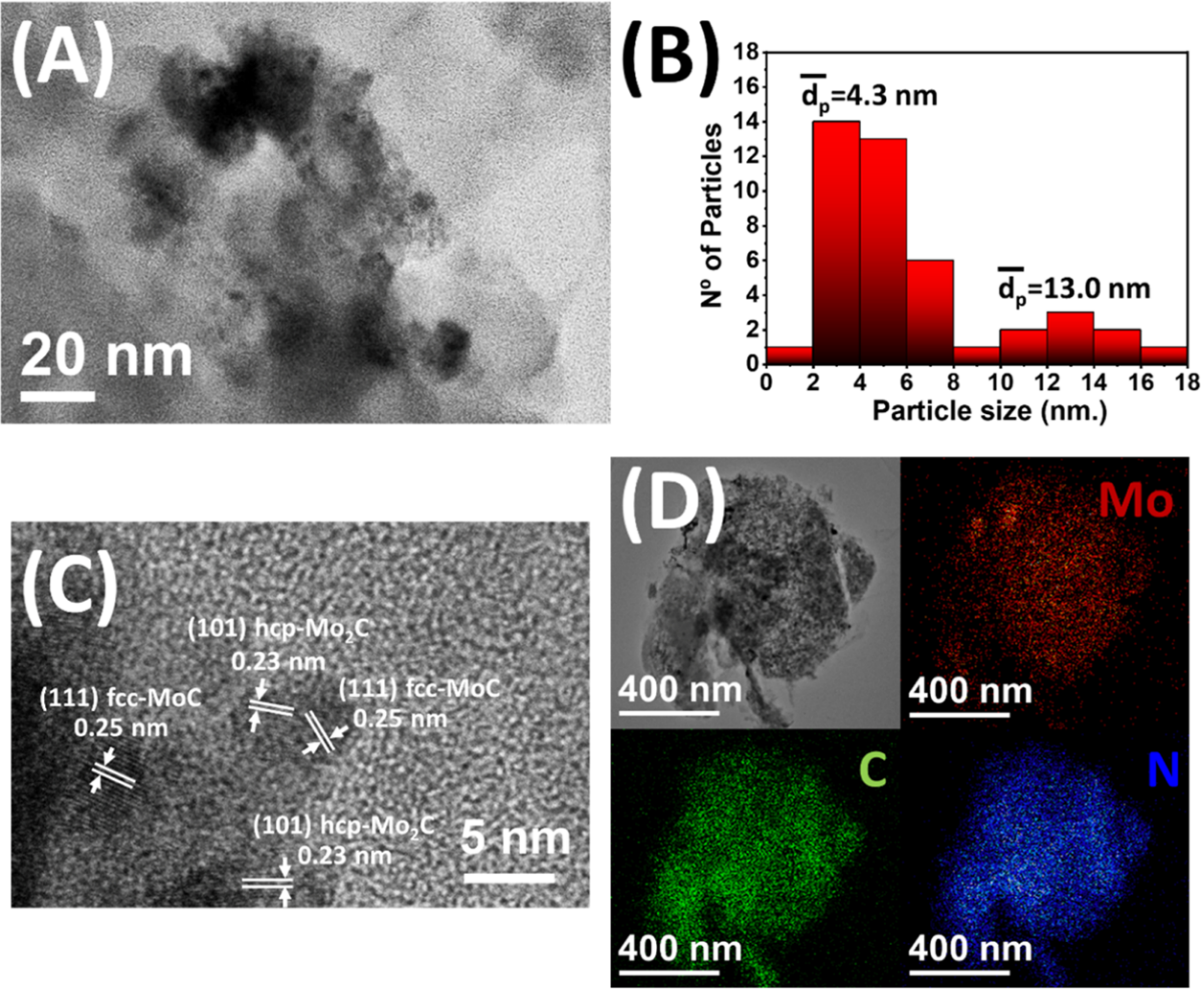
(A) TEM micrograph, (B) particle size distribution, (C) HRTEM micrograph, and (D) scanning transmission electron microscopy (STEM) image and EDX elemental mapping of the Mo_*x*_C700/g-C_3_N_4_ photocatalyst.

Figure 3 shows TEM and HRTEM images of Mo_*x*_C700/g-C_3_N_4_. In this case, the nanoparticles of Mo_*x*_C with two domains of particle size, with mean sizes of 4.3 and 13.0 nm, can be seen (Figure 3B). Moreover, the presence of hcp Mo_2_C and fcc MoC in close proximity was determined by HRTEM (Figure 3C). These results are in good agreement with those of the XRD analysis. Moreover, EDX analysis showed that Mo was homogeneously distributed along the photocatalyst (Figure 3D).

**Figure 4:**
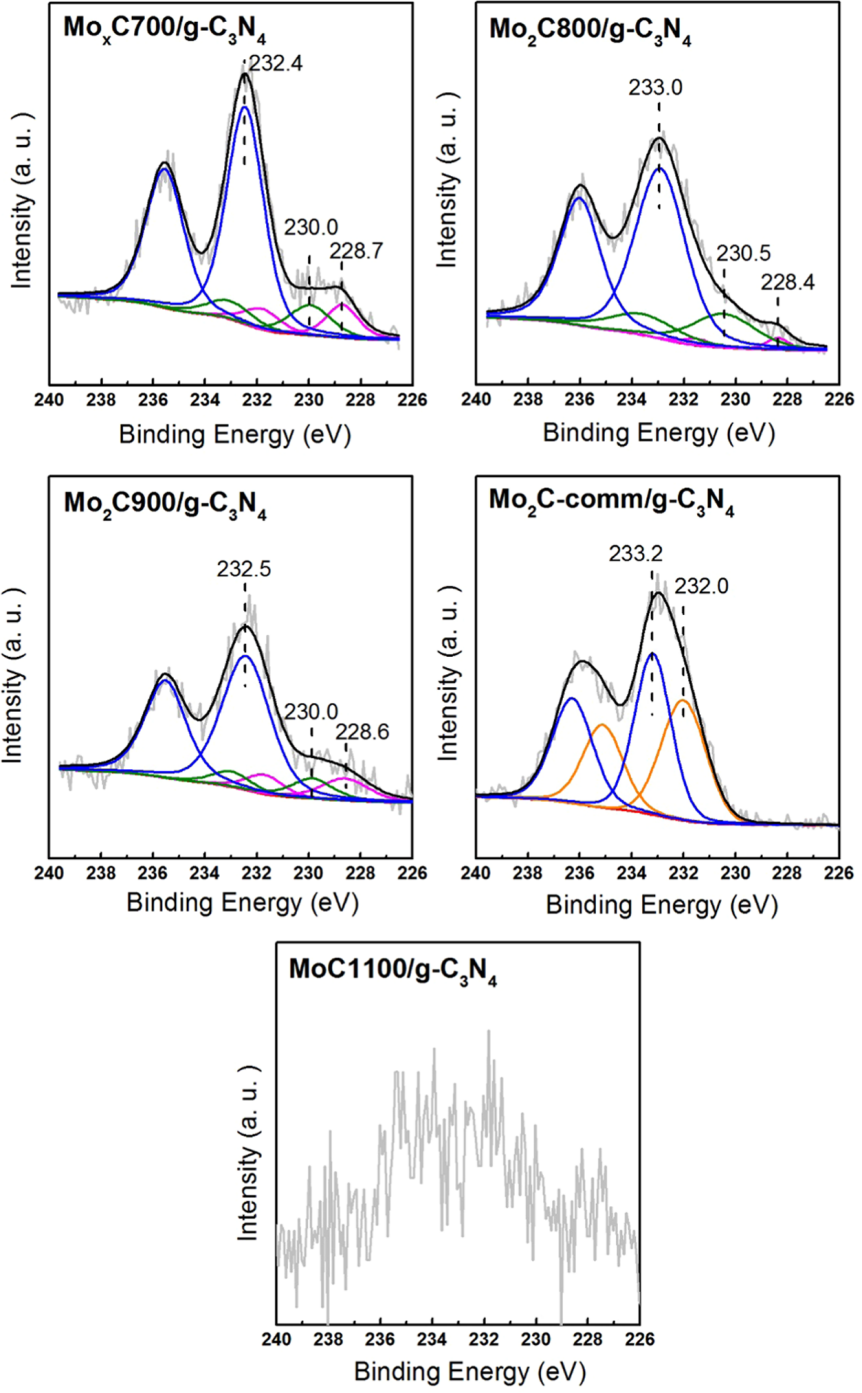
Mo 3d core level spectra of the nanocomposites.

The surface characteristics of the photocatalysts were analyzed by XPS. The XP C 1s spectrum corresponding to g-C_3_N_4_ (Figure S7) shows, besides the adventitious band due to C–C at 284.8 eV, a main band at about 288.1 eV, characteristic of bound C in the heterocycles (C–N=C).^40,48^ In addition, a small C 1s component at a lower BE (283.3–283.8 eV), characteristic of carbides can be identified in the XP spectra of Mo_*x*_C-containing photocatalysts (Figure S7).^24,37,38^ In all cases, the broadness and asymmetry of the band located at the highest BE are indicative of the presence of O-bound (C–O, C=O) surface species.^24,40,49^ The N 1s spectrum of g-C_3_N_4_ (Figure S7) shows a main component assigned to sp^2^-bonded N in the heterocycles at about 398.6 eV, and components at higher BEs of 399.4, 401.0, and 404.7 eV. The signal at 399.4 eV can be attributed to the presence of tertiary nitrogen (N-(C)_3_). On the other hand, amino functional groups (C–N–H), arising from a defective condensation of heptazine substructures, could be responsible for the component at 401.0 eV; the very low intense peak at 404.7 eV is attributed to the charging effects or positive charge localization in the heterocycles.^48,50,51^ The N 1s spectra of the nanocomposites were similar to those of g-C_3_N_4_ (Figure S7). On the other hand, the presence of a small amount of surface oxygen-containing species could be evidenced in all cases (Figure S8); the asymmetric broad O 1s band with a maximum at 532.6 eV in the XP spectrum of g-C_3_N_4_ could be assigned to adsorbed H_2_O and surface species with C=O bonds.^40^ Molybdenum oxide and/or oxycarbide species could contribute to the O 1s band in the XP spectra of Mo_*x*_C-containing photocatalysts, whose maxima are located at a lower BE than that of g-C_3_N_4_.^24,49^ Finally, Figure 4 shows the Mo 3d core level spectra of nanocomposites, which were deconvoluted fixing the Mo 3d_5/2_/Mo 3d_3/2_ intensity ratios of 1.5 and 3.1 eV as the value of orbital splitting.^52^ The Mo 3d_5/2_ component at 228.4–228.7 eV indicates the presence of surface carbides and is attributed to Mo_2_C and/or derived oxycarbide species.^24,37,49,53^ The other Mo 3d_5/2_ components at higher BEs can be assigned to the Mo^4+^, Mo^5+^, and Mo^6+^ surface species, which could be related to MoC and/or different molybdenum oxycarbide and oxide species.^37,39,53^ The samples Mo_*x*_C700/g-C_3_N_4_, Mo_*x*_C800/g-C_3_N_4_, and Mo_*x*_C900/g-C_3_N_4_ showed clear Mo 3d_5/2_ components at a BE lower than 231 eV, and the corresponding molybdenum surface species were about 23% of the total Mo^n+^ surface species. Although the Mo 3d spectrum of MoC1100/g-C_3_N_4_ could not be properly deconvoluted, the existence of surface molybdenum carbide and oxide species could also be inferred in this case (Figure 4). In all cases, the contact of samples with ambient air could produce oxycarbide and oxide species.^24^

### 3.2 Photoelectrochemical Features and Photocatalytic Behavior

The optical properties of the photocatalysts were analyzed by UV–vis eliminate DRS and PL spectroscopy. Figure S9 shows the UV–vis DRS spectra and the corresponding Tauc plots used in the determination of the band gap values, following the approach reported in ref (54). In all cases, light absorption edges occurred in the visible region (Table 1). The band gap determined for g-C_3_N_4_ is 2.75 eV, and all of the Mo_*x*_CT/g-C_3_N_4_ nanocomposites showed only slightly higher band gap values (2.77–2.79 eV).

**Figure 5:**
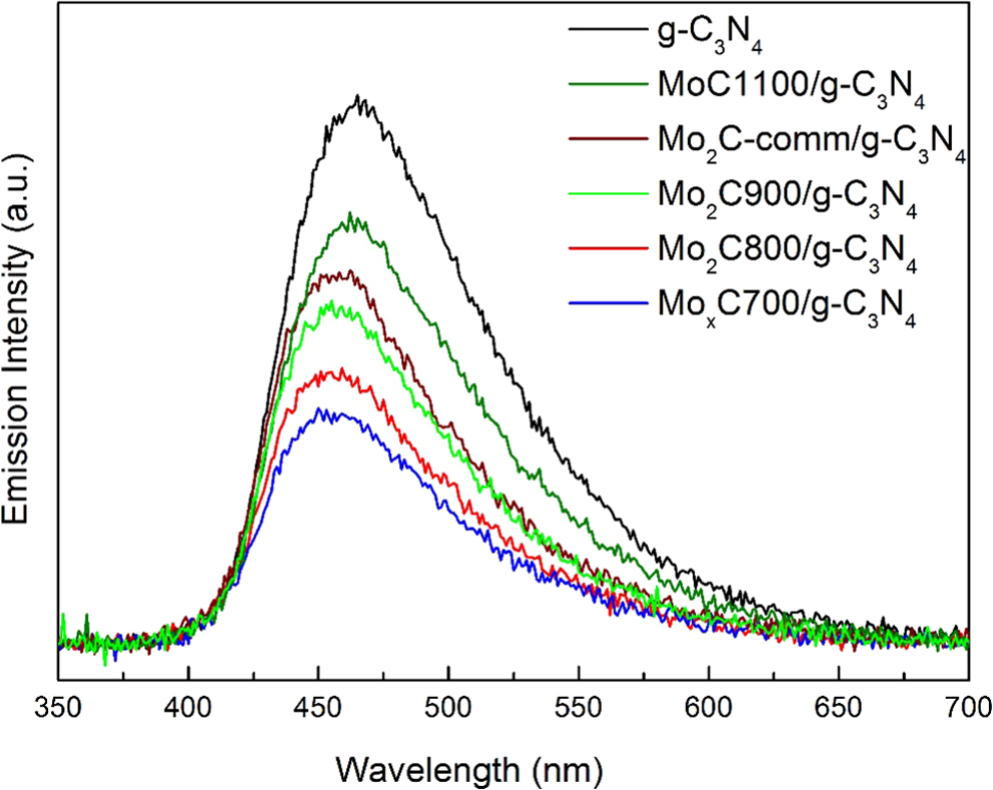
Photoluminescence spectra of nanocomposites and g-C_3_N_4_.

The recombination process of photogenerated (e^–^/h^+^) pairs in the photocatalysts was studied by PL analysis. Figure 5 shows the PL spectra of the nanocomposites compared to that of g-C_3_N_4_. We observed an emission peak in the visible region for all samples, a maximum at 465 nm for g-C_3_N_4_, and a slight shift of the emission maxima toward higher energies for the Mo_*x*_C-containing photocatalysts. These findings are consistent with the UV–vis DRS results, which indicate a slight increase in the band gap energy for Mo_*x*_CT/g-C_3_N_4_ than pristine g-C_3_N_4_. Interestingly, the intensity of the PL spectrum of g-C_3_N_4_ is always higher than that of nanocomposites (Figure 5), indicating that the presence of Mo_*x*_C decreases the e^–^/h^+^ pair recombination rate, thus leading to a decrease in the emission intensity. The trend in the intensity of the PL emission band (Figure 5) suggests that hcp Mo_2_C is more effective in reducing charge recombination in g-C_3_N_4_ than fcc MoC. Moreover, it seems that recombination is less favored when the Mo_2_C crystallite size decreases and when hcp Mo_2_C and fcc MoC are in close proximity over g-C_3_N_4_. A low recombination rate of the photogenerated (e^–^/h^+^) pairs and a faster electron transfer rate can improve the photocatalytic properties.

To further evaluate the photocharge separation and electron transfer properties of the photocatalysts, electrochemical impedance spectra, and transient photocurrent response were measured, as described in the Experimental Section.

**Figure 6:**
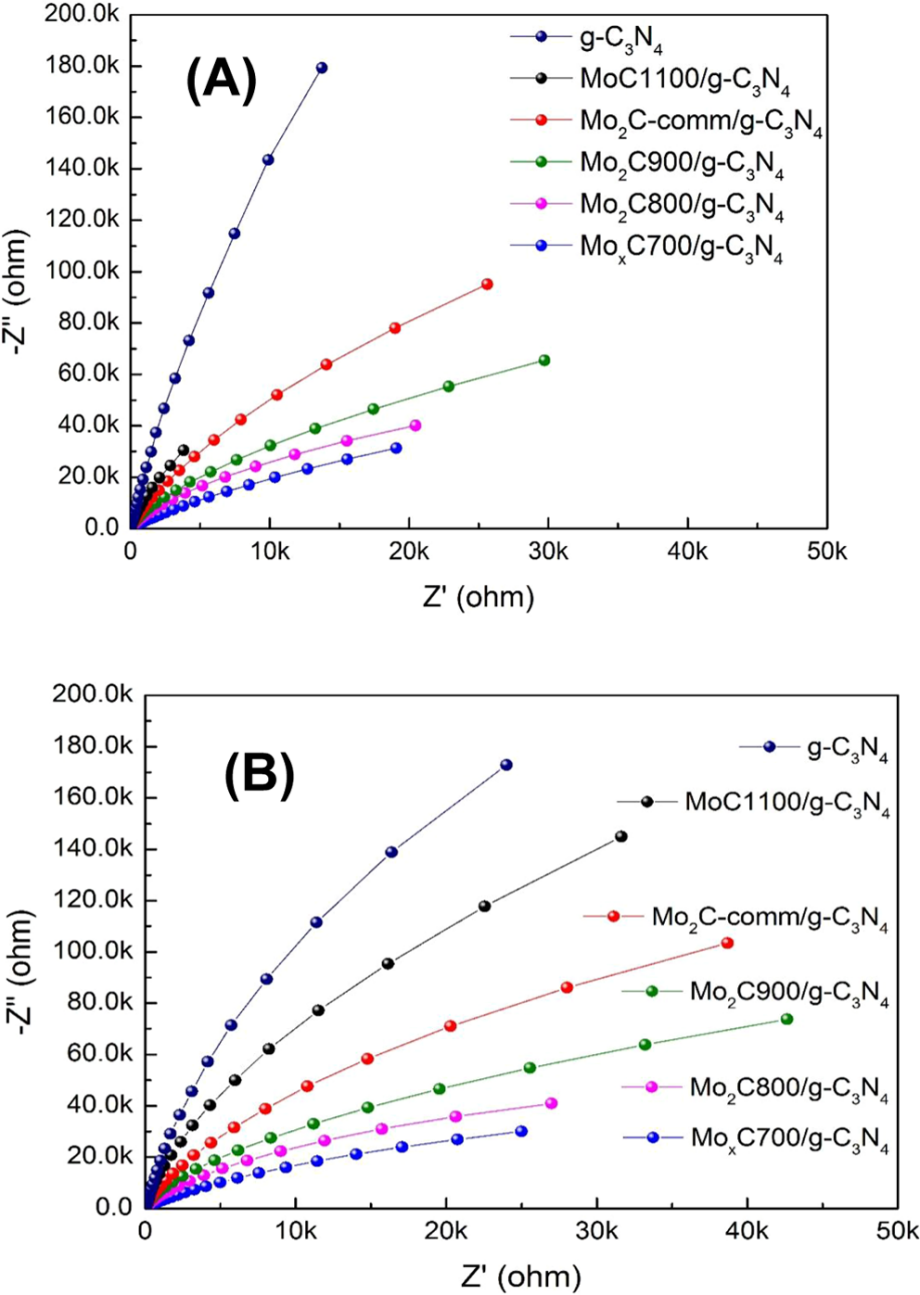
EIS Nyquist plots of nanocomposites and g-C_3_N_4_: (A) under dark conditions and (B) with irradiation.

Figure 6 shows the impedance Nyquist plots for the g-C_3_N_4_ and Mo_*x*_CT/g-C_3_N_4_ photocatalysts under dark (A) and illumination (B) conditions. As can be seen, in both cases, g-C_3_N_4_ shows a larger arc radius than the Mo_*x*_C-containing nanocomposites, indicating that the presence of Mo_*x*_C on the g-C_3_N_4_ nanosheets reduces the electron transfer resistance in the dark and under irradiation. Moreover, in all cases, the arc radius in the dark is larger than that under irradiation, as illustrated in Figure S10 for the samples with the largest and the smallest arc radii, g-C_3_N_4_ and Mo_*x*_C700/g-C_3_N_4_, respectively. This indicates the effectiveness of irradiation in decreasing the barrier of electron transfer in these materials. The Nyquist arc radius follows a trend similar to that of the intensity of the PL spectra shown above; the lower the electron transfer barrier, the higher the efficiency of charge separation.

**Figure 7:**
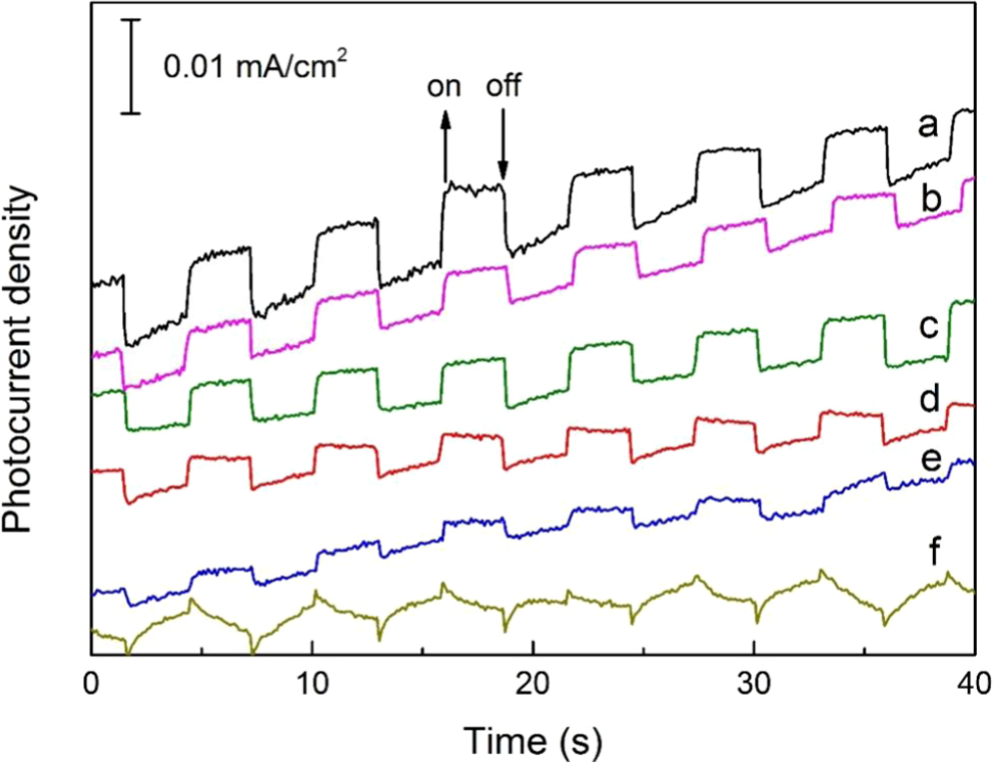
Transient photocurrent responses of nanocomposites and g-C_3_N_4_: (a) Mo_*x*_C700/g-C_3_N_4_; (b) Mo_2_C800/g-C_3_N_4_; (c) Mo_2_C900/g-C_3_N_4_; (d) Mo_2_C-comm/g-C_3_N_4_; (e) MoC1100/g-C_3_N_4_; and (f) g-C_3_N_4_.

The results of the transient photocurrent responses are shown in Figure 7. The Mo_*x*_C-containing nanocomposites showed a larger photocurrent density than that of g-C_3_N_4_. Mo_*x*_C700/g-C_3_N_4_ showed the highest photocurrent density. The presence of Mo_*x*_C as a cocatalyst slows down the recombination rate of photogenerated e^–^/h^+^, decreases the barrier for electron transport, and accordlingly can favor the further transfer of electrons for proton reduction.

**Figure 8:**
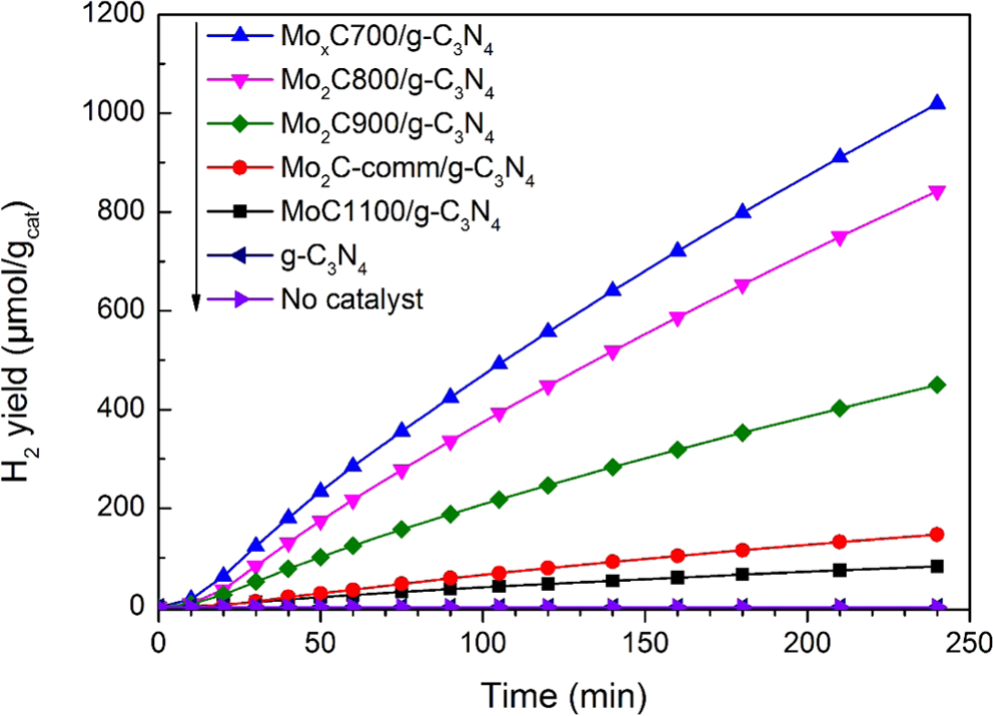
H_2_ generated per gram of photocatalyst as a function of irradiation time; results from g-C_3_N_4_ and the blank test are also included. Reaction conditions: 25% v/v ethanol aqueous solution and *T* = 20 °C.

As stated above, the photocatalytic behavior in H_2_ generation from EtOH_aq_ (25% v/v) was analyzed under visible light (λ > 385 nm) for all of the materials prepared, including pristine g-C_3_N_4_, and a blank test without the photocatalyst was also carried out. Under the experimental conditions used in this work, g-C_3_N_4_ and the blank test produced a negligible amount of H_2_. On the other hand, all nanocomposites containing molybdenum carbide species were active in photocatalytic H_2_ generation; the H_2_ production per gram of catalyst over time is depicted in Figure 8. Besides H_2_, minor amounts of CO_2_ were produced; moreover, mainly 2,3-butanediol was found in the liquid phase, and no O_2_ was detected. These results point out that photoreforming of ethanol was not accomplished. Moreover, the absence of CH_4_ and CO as products in the gas phase suggests that under our experimental conditions and withMo_*x*_C/g-C_3_N_4_ photocatalysts, the cleavage of the C–C bond is not favored in the ethanol phototransformation.^1,2,4^ The oxidation of ethanol could proceed with the generated hole (h^+^), and the α-hydroxyethyl radicals (^•^CH(OH)CH_3_) be formed:^38,55,56^
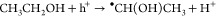
1Then, the coupling of two initially formed α-hydroxyethyl radicals (^•^CH(OH)CH_3_) could be proposed for the formation of 2,3-butanediol:^38,44,56,57^
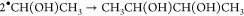
2

According to the characterization results given above, the molybdenum carbide species on g-C_3_N_4_ mainly facilitate electronic transfer, producing H_2_ and decreasing e^–^/h^+^ recombination in g-C_3_N_4_. This is illustrated in Figure S11 for Mo_*x*_C700/g-C_3_N_4_.

Figure 8 shows the hydrogen production per gram of the Mo_*x*_CT/g-C_3_N_4_ photocatalyst; a similar trend is found when H_2_ production is referred to as a gram of Mo (Figure S12). The photocatalytic performance can be straightforwardly related to the photoelectrochemical characteristics of the samples: the intensity of the PL emission peak, EIS values, and transient photocurrent measurements. The smaller the size of the hcp Mo_2_C particles in the nanocomposite, the better the photocatalytic performance. Moreover, a positive effect of the close proximity of the hcp Mo_2_C and fcc MoC phases is found. Mo_*x*_C700/g-C_3_N_4_ with both hcp Mo_2_C (11 nm) and fcc MoC (4 nm) showed the best photocatalytic behavior. The presence of heterojunctions MoC-Mo_2_C could improve the photocatalytic behavior, as has been demonstrated for WC-Mo_2_C/TiO_2_ photocatalysts with WC-Mo_2_C heterojunctions.^58^ In fact, as stated in the Introduction section, for Mo_*x*_C-based electrocatalysts, the presence of MoC/Mo_2_C heterostructures was demonstrated to improve the H_2_ evolution reaction,^36^ and an improved photocatalytic behavior for H_2_ production could be expected,^35^ which is in line with the results presented here.

**Figure 9:**
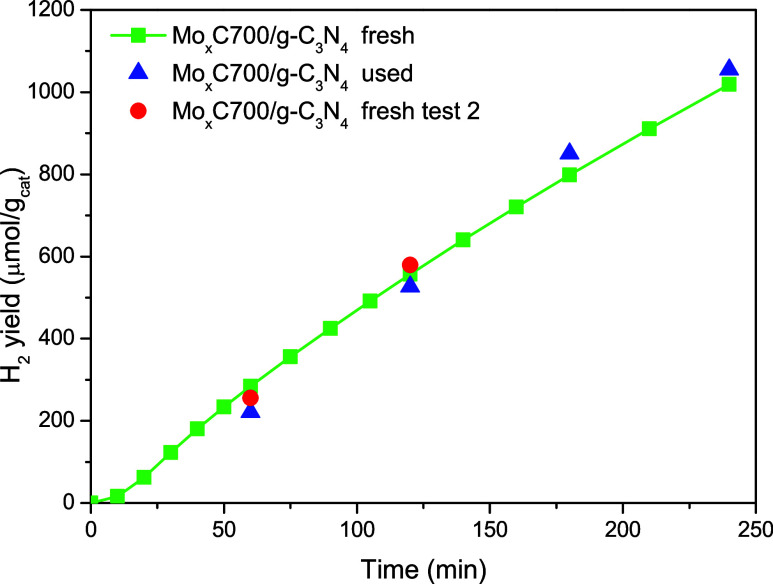
Different photocatalytic tests for H_2_ production over fresh and used Mo_*x*_C700/g-C_3_N_4_. In test 2, the light was switched off after 1 h; then, after 0.5 h in dark conditions, the light was switched on. Reaction conditions: 25% v/v ethanol aqueous solution and *T* = 20 °C.

In order to know the reusability of the most performant photocatalyst, a new photocatalytic test was carried out with the used Mo_*x*_C700/g-C_3_N_4_ (Figure 9), and a similar H_2_ production for both fresh and used catalysts was observed. Figure 9 also shows the reproducibility of the photocatalytic behavior of fresh Mo_*x*_C700/g-C_3_N_4_ when the light switch off/switch on procedure was employed. In all cases, the standard deviation was only up to 2% of the values originally obtained. The reused Mo_*x*_C700/g-C_3_N_4_ was characterized by FTIR and XRD, and similar results were obtained for the fresh photocatalysts (Figures S13 and S14). These results show the stability of Mo_*x*_C700/g-C_3_N_4_, which can be reused without loss of its properties.

## 4 Conclusions

The preparation method led to tailored Mo_*x*_CT/g-C_3_N_4_ photocatalysts with hcp Mo_2_C and/or fcc MoC nanoparticles of different sizes supported on g-C_3_N_4_ nanosheets. The separately prepared Mo_*x*_C cocatalysts were incorporated into g-C_3_N_4_ using ultrasound, which maintained the characteristics of both Mo_*x*_C nanoparticles and g-C_3_N_4_ nanosheets formerly synthesized.

The photocatalytic behavior of Mo_*x*_CT/g-C_3_N_4_ in hydrogen generation from EtOH_aq_ under visible light is related to the rate of recombination of photogenerated charges, electron transfer resistance, and photocurrent response of the prepared nanocomposites. These properties depend on the characteristics of the Mo_*x*_C cocatalyst. Photocatalysts containing hcp Mo_2_C were more effective than that containing only fcc MoC; the smaller the crystallite size of hcp Mo_2_C, the better was the photocatalytic performance of Mo_*x*_CT/g-C_3_N_4_. The best photocatalytic results were obtained for Mo_*x*_C700/g-C_3_N_4_, which presented hexagonal Mo_2_C and cubic MoC, the lowest rate of charge recombination and electron transfer resistance, and the highest photocurrent response; about 7 mmol of H_2_ g_Mo_^–1^ h^–1^ were produced with this photocatalyst under the conditions used. The improved photocatalytic behavior of Mo_*x*_C700/g-C_3_N_4_ could be related to the presence of MoC-Mo_2_C heterojunctions, which could enhance the photoelectrochemical properties of the nanocomposites and therefore photocatalytic H_2_ generation. Moreover, the reusability of Mo_*x*_C700/g-C_3_N_4_ is demonstrated.

## Supporting Information Available

Additional details related to characterization methods and characterization and photocatalytic results are included (PDF)
