# Supplementary material for: MoxC Heterostructures as Efficient Cocatalysts in Robust MoxC/g-C3N4 Nanocomposites for Photocatalytic H2 Production from Ethanol
Source: ACS Sustain Chem Eng. 2024 Mar 7;12(11):4365–74. doi: 10.1021/acssuschemeng.3c06261 (PMC10954046; doi:10.1021/acssuschemeng.3c06261)
Supplement: Supplementary file 1 — sc3c06261_si_001.pdf [file sc3c06261_si_001.pdf]

Supplementary Information

Mo_xC heterostructures as efficient co-catalysts in robust $Mo_xC/g-C_3N_4$ nanocomposites for photocatalytic H_2 production from ethanol

Yan Wang[1,2,&], Arturo Pajares[1,2,#], Jarosław Serafin[1], Xavier Alcobé[3], Frank Güell[4], Narcís Homs[1,2,*], Pilar Ramírez de la Piscina[1]

[1]*Departament de Química Inorgànica i Orgànica, secció de Química Inorgànica & Institut de Nanociència i Nanotecnologia (IN2UB), Universitat de Barcelona, Martí i Franquès 1, 08028 Barcelona, Spain.*

[2]*Catalonia Institute for Energy Research (IREC), Jardins de les Dones de Negre 1, 08930 Barcelona, Spain.*

[3]*Unitat de Difracció de Raigs X, Centres Científics i Tecnològics (CCiTUB), Universitat de Barcelona, Lluís Solé i Sabaris 1-3, 08028 Barcelona, Spain.*

[4]*ENPHOCAMAT Group, Institut de Nanociència i Nanotecnologia (IN2UB), Universitat de Barcelona, Martí i Franquès 1, 08028 Barcelona, Spain.*

[&]*Present address: Shenzhen Institute of Advanced Technology, Chinese Academy of Sciences, 1068 Xueyuan Avenue, Shenzhen University Town, Shenzhen, China.*

[#]*Present address: Sustainable Materials Management, Flemish Institute for Technological Research (VITO NV), Boeretang 200, 2400 Mol, Belgium*

(*) Corresponding author: narcis.homs@qi.ub.edu

Number of pages: 14

Number of figures: 14 [**Figures S1-S14**]

Number of tables: 0

References: 1

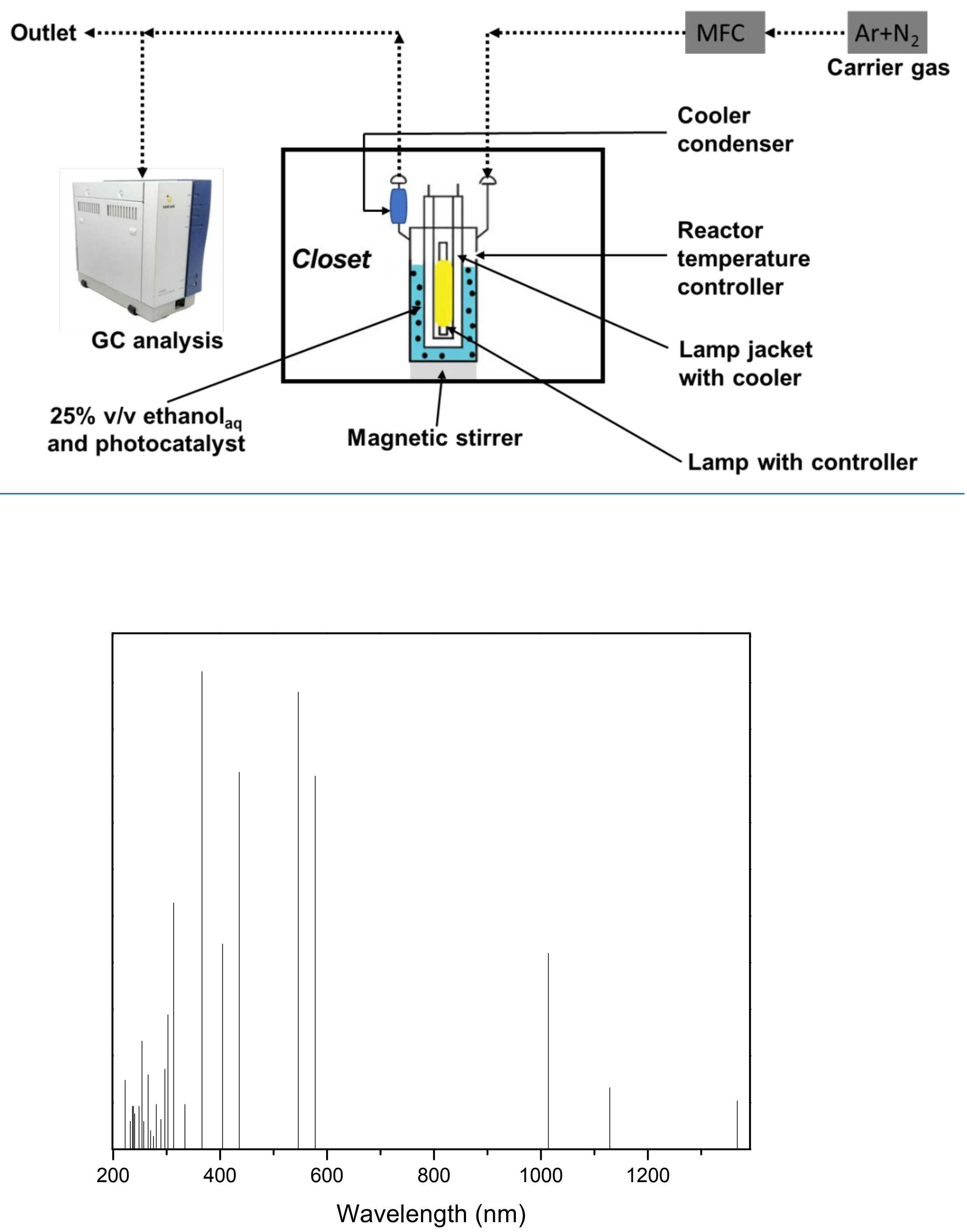

Figure S1. Schematic diagram of the setup used to carry out the photocatalytic experiments; on the bottom, emission lines of the lamp used for irradiation.

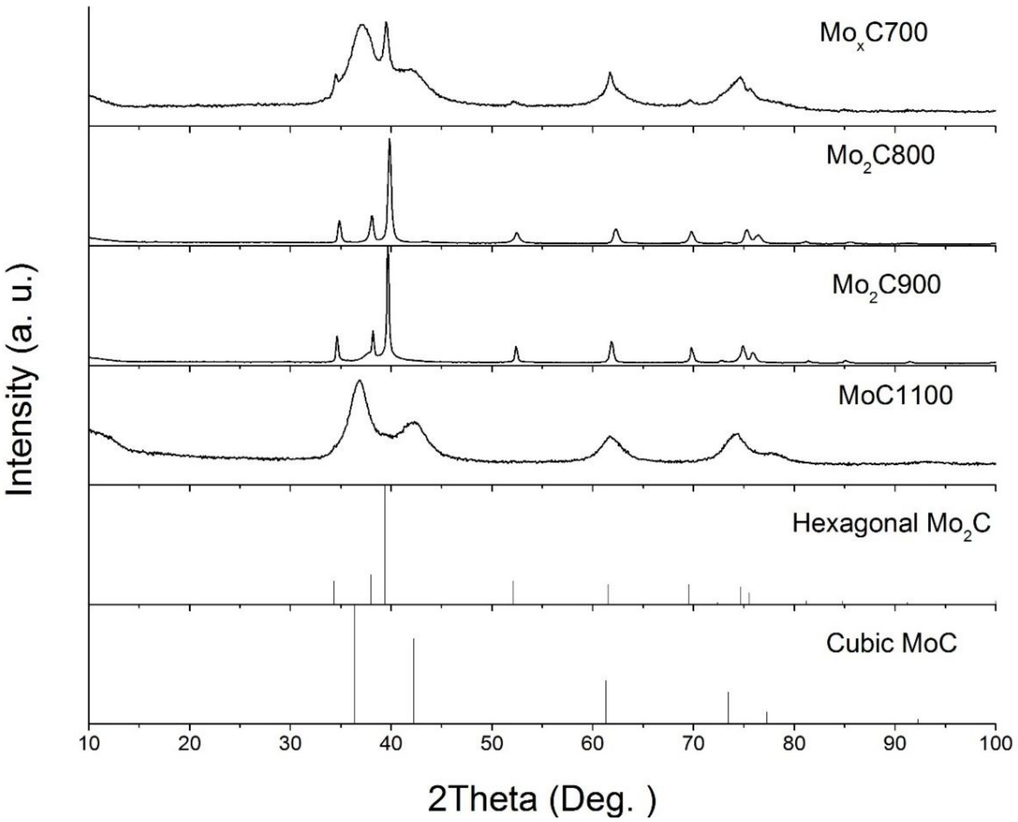

Figure S2. XRD patterns of Mo_xCT materials used in the preparation of $Mo_xCT/g\text{-}C_3N_4$ photocatalysts.

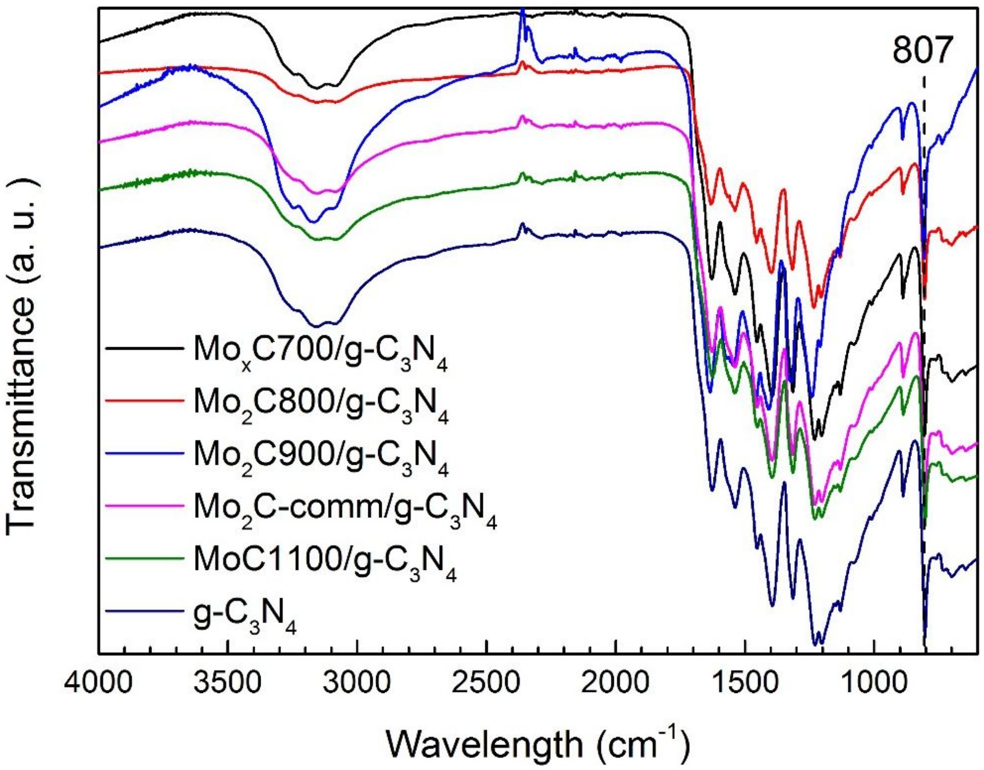

Figure S3. FTIR spectra of nanocomposites and $g-C_3N_4$.

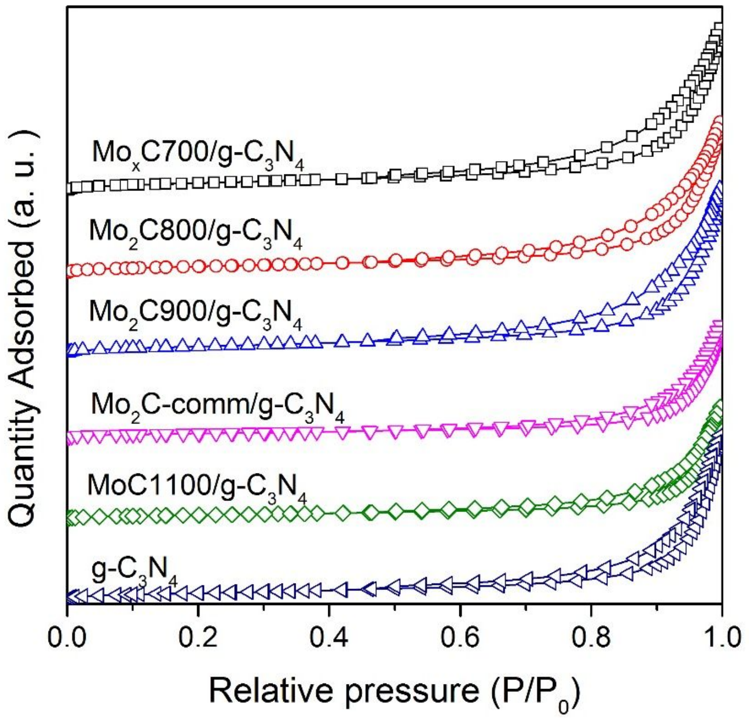

Figure S4. N_2 adsorption-desorption isotherms of nanocomposites and g-C_3N_4.

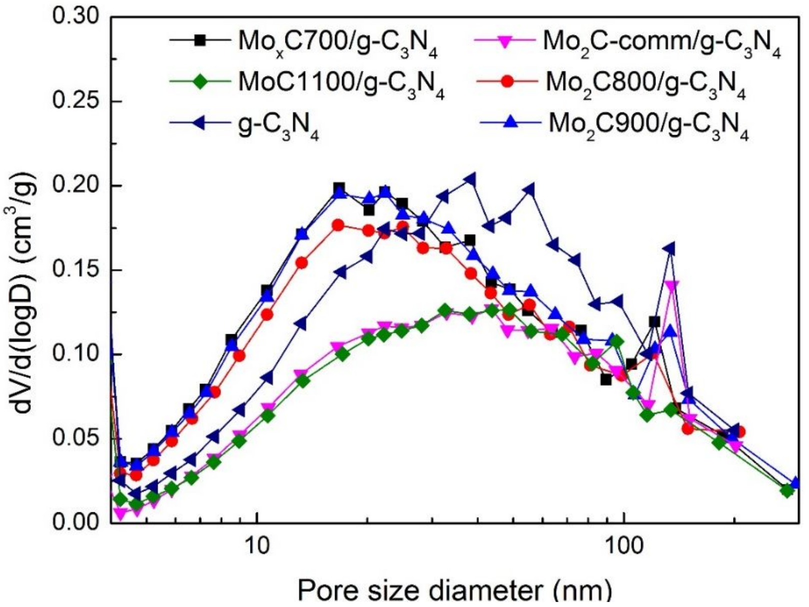

Figure S5. Pore size distribution of nanocomposites and g-C_3N_4.

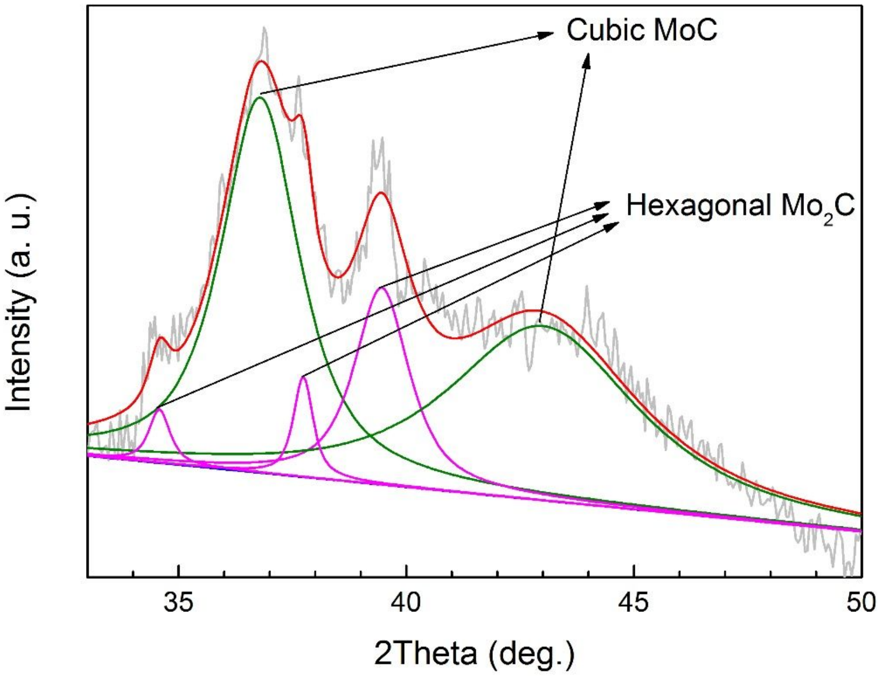

Figure S6. XRD pattern of $Mo_xC700/g\text{-}C_3N_4$ and full profile analysis in the 30-50º 2θ range, used for the calculation of crystallite size of hcp Mo_2C and fcc MoC (Table 1).

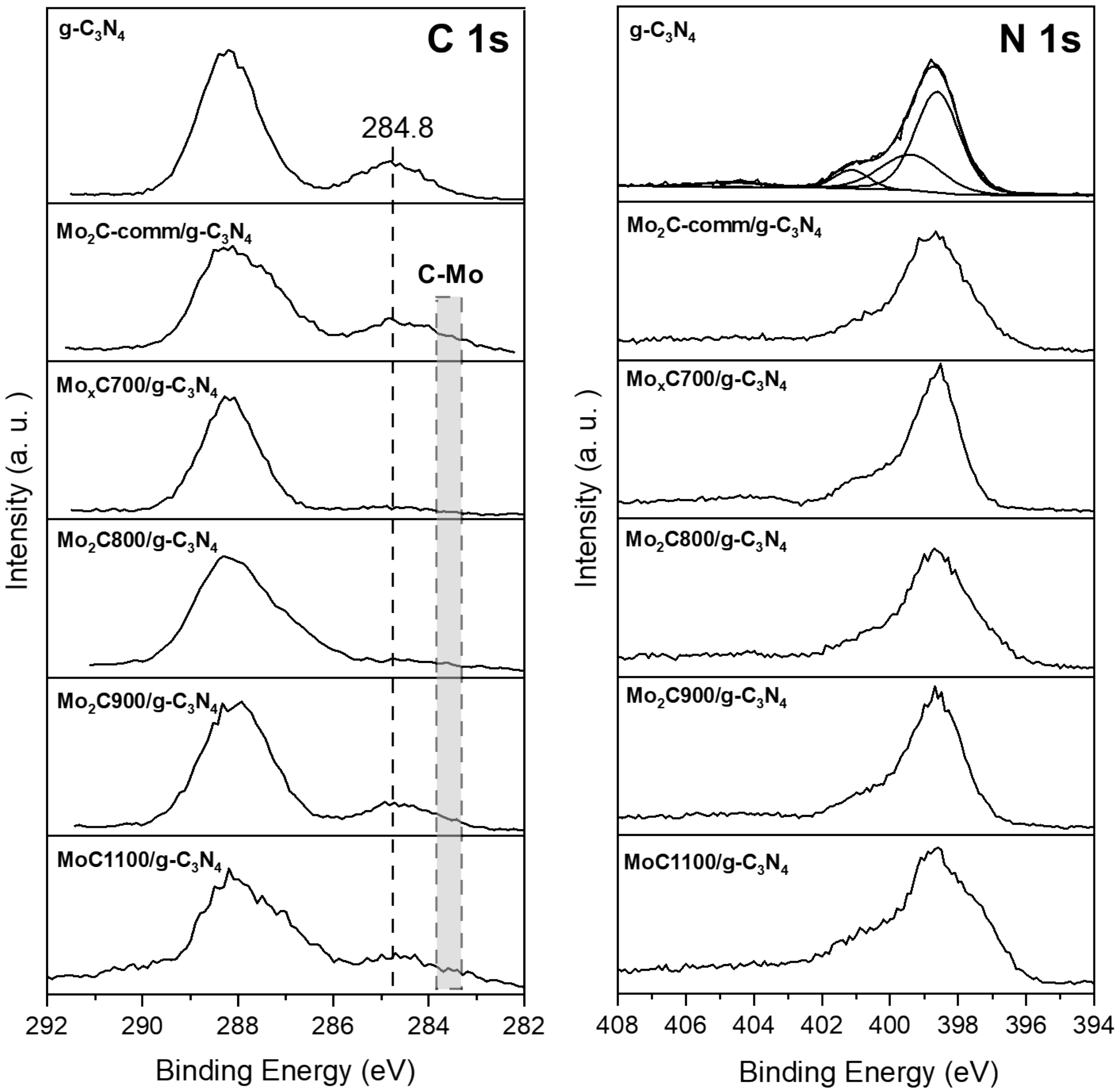

Figure S7. C 1s and N 1s core level spectra of nanocomposites and g-C_3N_4.

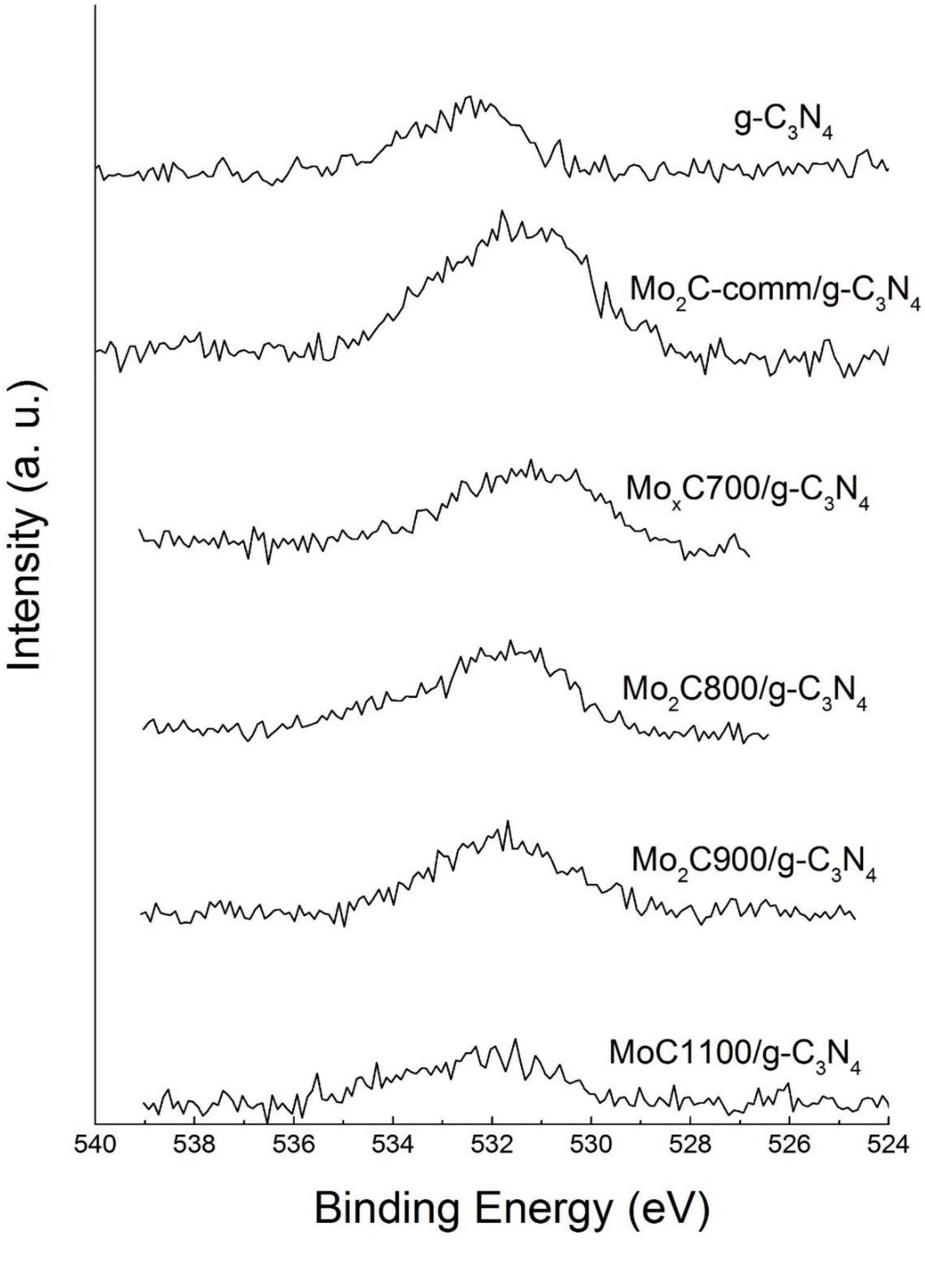

Figure S8. O 1s core level spectra of nanocomposites and $g\text{-}C_3N_4$.

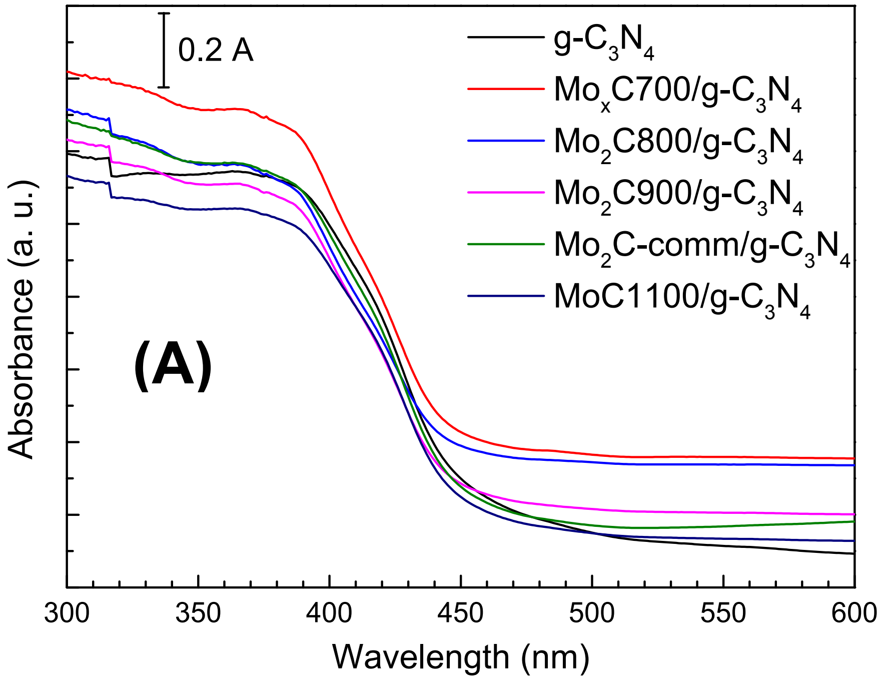

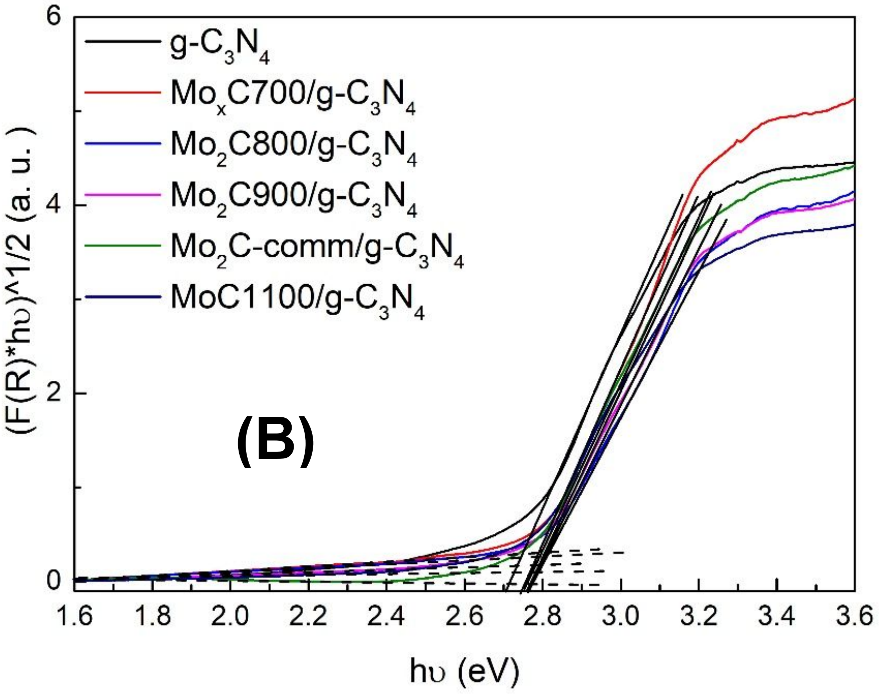

Figure S9. A) UV–vis diffuse reflectance spectra and, B) Tauc plots of the Kubelka-Munk function versus photon energy (hυ) of nanocomposites and g-C_3N_4. The plots were used for the calculation of band gap values in Table 1, using the intersection between the linear fit for the slope before the fundamental absorption and the linear fit of the Tauc plot.[1]

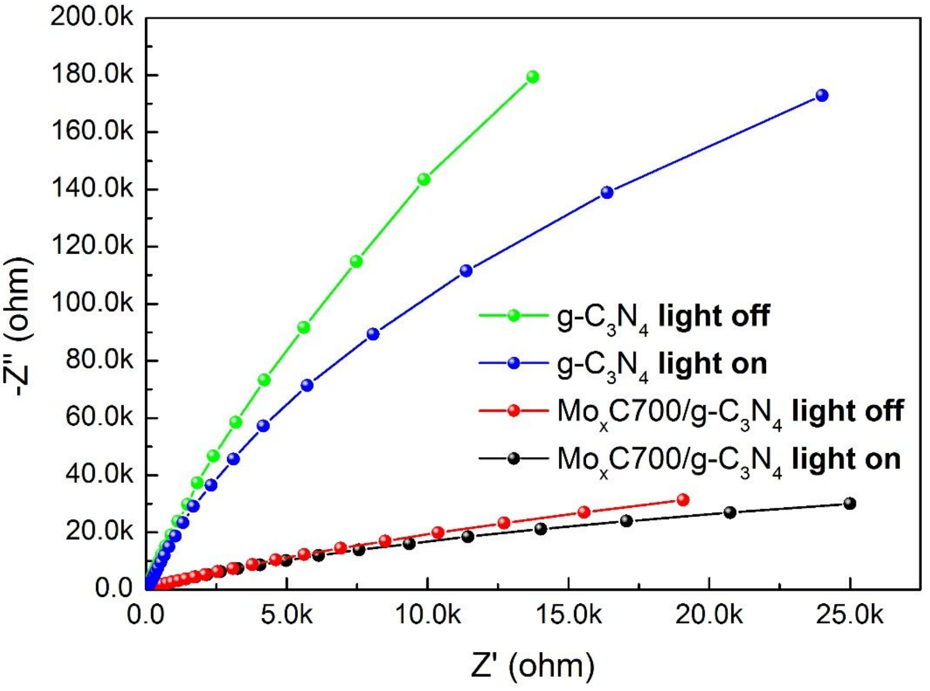

Figure S10. EIS Nyquist plots of Mo_xC700/g-C_3N_4 and g-C_3N_4 in dark and under irradiation.

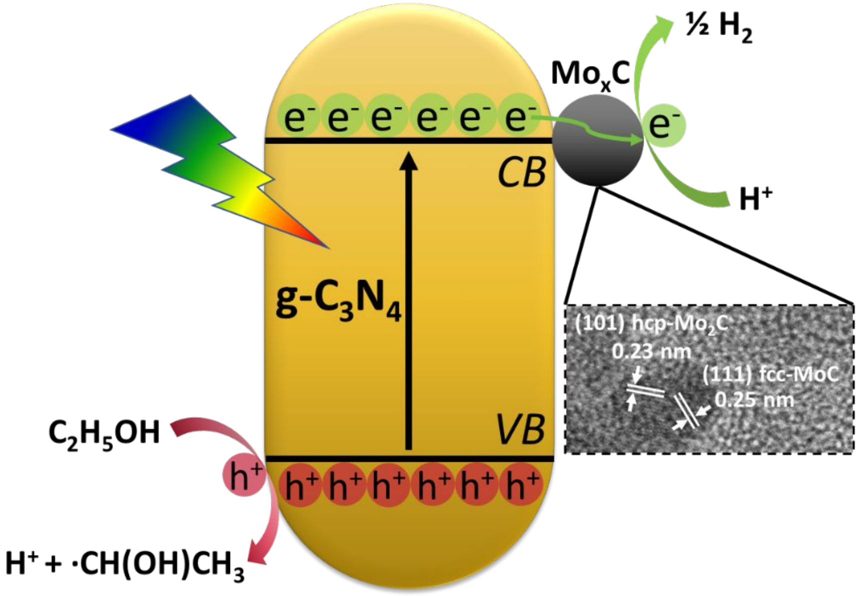

Figure S11. Schematic diagram of the photocatalytic H_2 production over $Mo_xC/g\text{-}C_3N_4$ nanocomposites, illustrated for $Mo_x700/g\text{-}C_3N_4$.

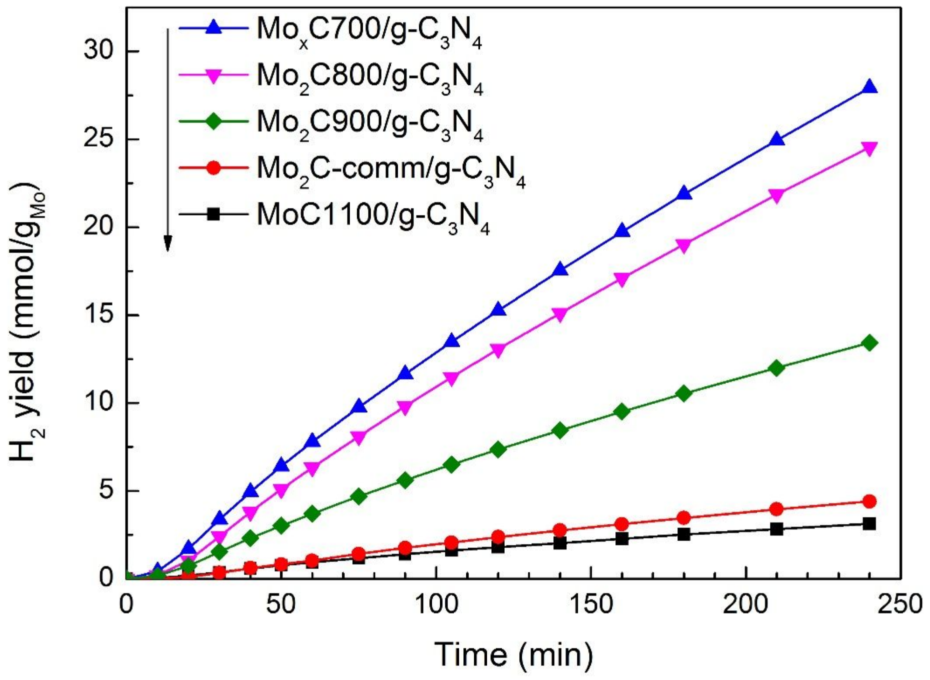

Figure S12. H_2 produced per gram of Mo as a function of irradiation time over nanocomposites. Reaction conditions: EtOH (25% v/v), T=20 ºC.

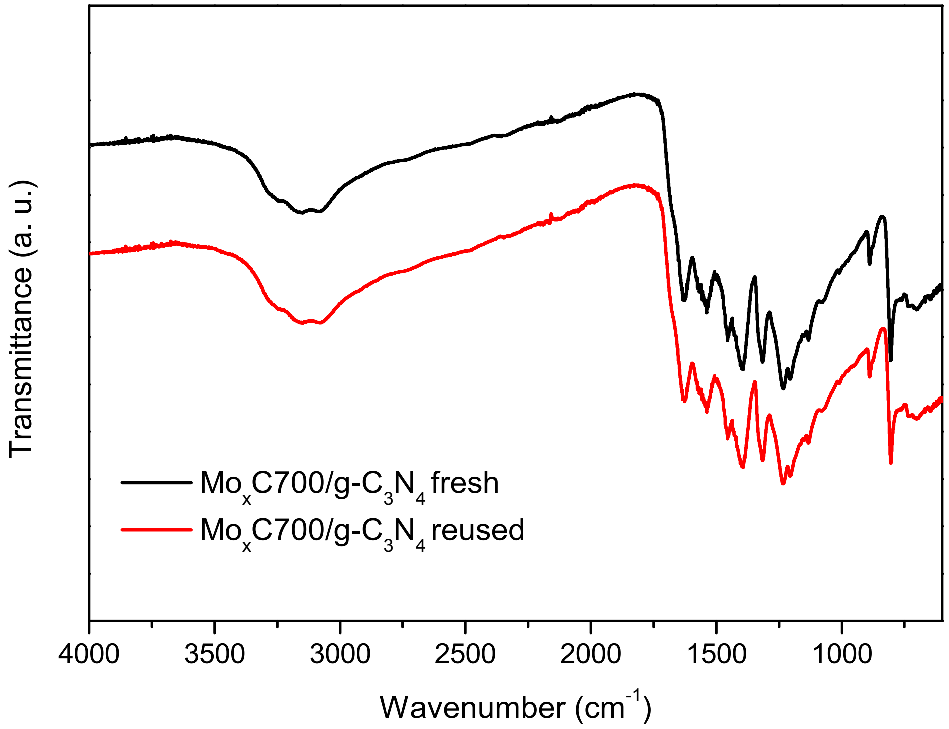

Figure S13. FTIR spectra of fresh and re-used $Mo_xC700/g\text{-}C_3N_4$.

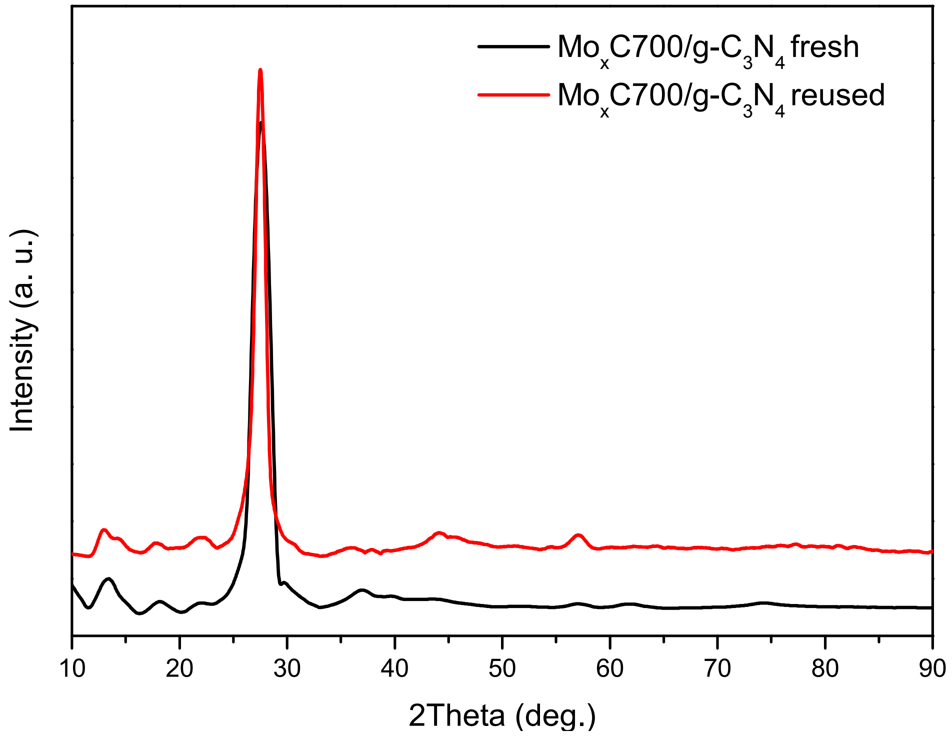

Figure S14. XRD patterns of fresh and re-used $Mo_xC700/g\text{-}C_3N_4$.

References

[1] Makuła, P.; Pacia, M.; Macyk, W. How To Correctly Determine the Band Gap Energy of Modified Semiconductor Photocatalysts Based on UV-Vis Spectra. *J. Phys. Chem. Lett.* **2018**, *9* (23), 6814-6817. https://doi.org/10.1021/acs.jpclett.8b02892
